# Supplementary material for: Multi‐dataset identification of innovative feature genes and molecular mechanisms in keratoconus
Source: J Cell Mol Med. 2024 Sep 19;28(18):e70079. doi: 10.1111/jcmm.70079 (PMC11412914; doi:10.1111/jcmm.70079)
Supplement: Supplementary file 2 — Material S2. [file JCMM-28-e70079-s002.zip › 7.GSEA analysis/1.ARL11/KEGG_ALDOSTERONE_REGULATED_SODIUM_REABSORPTION.html]

Details for gene set KEGG\_ALDOSTERONE\_REGULATED\_SODIUM\_REABSORPTION[GSEA]

|  || Dataset | ARL11.group.cls#h\_versus\_l |
| Phenotype | group.cls#h\_versus\_l |
| Upregulated in class | l |
| GeneSet | KEGG\_ALDOSTERONE\_REGULATED\_SODIUM\_REABSORPTION |
| Enrichment Score (ES) | -0.29134387 |
| Normalized Enrichment Score (NES) | -0.9604487 |
| Nominal p-value | 0.48466256 |
| FDR q-value | 1.0 |
| FWER p-Value | 1.0 |
Table: GSEA Results Summary

  

Fig 1: Enrichment plot: KEGG\_ALDOSTERONE\_REGULATED\_SODIUM\_REABSORPTION      
 Profile of the Running ES Score & Positions of GeneSet Members on the Rank Ordered List

  

| SYMBOL | TITLE | RANK IN GENE LIST | RANK METRIC SCORE | RUNNING ES | CORE ENRICHMENT || 1 | PIK3R3 | na | 2180 | 0.284 | -0.0004 | No |
| 2 | ATP1B4 | na | 2566 | 0.261 | 0.0460 | No |
| 3 | PIK3CG | na | 2870 | 0.247 | 0.0916 | No |
| 4 | PIK3R1 | na | 6079 | 0.135 | 0.0289 | No |
| 5 | ATP1B1 | na | 6792 | 0.117 | 0.0340 | No |
| 6 | SLC9A3R2 | na | 7487 | 0.102 | 0.0365 | No |
| 7 | PIK3R2 | na | 8783 | 0.078 | 0.0164 | No |
| 8 | SCNN1A | na | 9359 | 0.069 | 0.0151 | No |
| 9 | INS | na | 9462 | 0.068 | 0.0271 | No |
| 10 | INSR | na | 10038 | 0.060 | 0.0237 | No |
| 11 | PIK3R5 | na | 10118 | 0.059 | 0.0344 | No |
| 12 | PRKCA | na | 10617 | 0.052 | 0.0315 | No |
| 13 | KRAS | na | 10630 | 0.052 | 0.0426 | No |
| 14 | PDPK1 | na | 10673 | 0.052 | 0.0528 | No |
| 15 | NEDD4L | na | 11021 | 0.047 | 0.0532 | No |
| 16 | FXYD4 | na | 13179 | 0.025 | -0.0034 | No |
| 17 | SGK1 | na | 13238 | 0.025 | 0.0004 | No |
| 18 | PIK3CA | na | 13512 | 0.023 | -0.0024 | No |
| 19 | ATP1A1 | na | 13911 | 0.020 | -0.0096 | No |
| 20 | PRKCG | na | 17545 | -0.007 | -0.1128 | No |
| 21 | IGF1 | na | 18329 | -0.013 | -0.1326 | No |
| 22 | ATP1B3 | na | 19840 | -0.023 | -0.1711 | No |
| 23 | SFN | na | 20070 | -0.024 | -0.1724 | No |
| 24 | ATP1A3 | na | 20328 | -0.026 | -0.1741 | No |
| 25 | FXYD2 | na | 22512 | -0.045 | -0.2270 | No |
| 26 | IRS4 | na | 22518 | -0.045 | -0.2172 | No |
| 27 | MAPK3 | na | 22614 | -0.046 | -0.2098 | No |
| 28 | SCNN1B | na | 22657 | -0.047 | -0.2007 | No |
| 29 | ATP1A4 | na | 22955 | -0.049 | -0.1984 | No |
| 30 | ATP1B2 | na | 23314 | -0.054 | -0.1969 | No |
| 31 | HSD11B2 | na | 23419 | -0.055 | -0.1878 | No |
| 32 | PIK3CB | na | 23818 | -0.060 | -0.1861 | No |
| 33 | MAPK1 | na | 24626 | -0.071 | -0.1937 | No |
| 34 | PIK3CD | na | 25759 | -0.087 | -0.2073 | No |
| 35 | NR3C2 | na | 26070 | -0.091 | -0.1961 | No |
| 36 | ATP1A2 | na | 29374 | -0.168 | -0.2543 | Yes |
| 37 | IRS2 | na | 29852 | -0.183 | -0.2278 | Yes |
| 38 | KCNJ1 | na | 30425 | -0.202 | -0.1999 | Yes |
| 39 | IRS1 | na | 32036 | -0.261 | -0.1888 | Yes |
| 40 | HSD11B1 | na | 33227 | -0.339 | -0.1485 | Yes |
| 41 | PRKCB | na | 33863 | -0.399 | -0.0790 | Yes |
| 42 | SCNN1G | na | 34293 | -0.472 | 0.0125 | Yes |
Table: GSEA details [plain text format]

  

Fig 2: KEGG\_ALDOSTERONE\_REGULATED\_SODIUM\_REABSORPTION      
 Blue-Pink O' Gram in the Space of the Analyzed GeneSet

  

Fig 3: KEGG\_ALDOSTERONE\_REGULATED\_SODIUM\_REABSORPTION: Random ES distribution      
 Gene set null distribution of ES for **KEGG\_ALDOSTERONE\_REGULATED\_SODIUM\_REABSORPTION**
